# Supplementary material for: Raman micro-spectroscopy reveals the spatial distribution of fumarate in cells and tissues
Source: Nat Commun. 2024 Jun 25;15:5386. doi: 10.1038/s41467-024-49403-w (PMC11199670; doi:10.1038/s41467-024-49403-w)
Supplement: Supplementary file 2 — Reporting Summary [file 41467_2024_49403_MOESM2_ESM.pdf]

nature portfolio

Corresponding author(s): S.E. Bohndiek, C. Frezza

Last updated by author(s): May 17, 2024

Reporting Summary

Nature Portfolio wishes to improve the reproducibility of the work that we publish. This form provides structure for consistency and transparency in reporting. For further information on Nature Portfolio policies, see our Editorial Policies and the Editorial Policy Checklist.

Statistics

For all statistical analyses, confirm that the following items are present in the figure legend, table legend, main text, or Methods section.

n/a	Confirmed	
☐	☒	The exact sample size (*n*) for each experimental group/condition, given as a discrete number and unit of measurement
☐	☒	A statement on whether measurements were taken from distinct samples or whether the same sample was measured repeatedly
☐	☒	The statistical test(s) used AND whether they are one- or two-sided<br>*Only common tests should be described solely by name; describe more complex techniques in the Methods section.*
☒	☐	A description of all covariates tested
☐	☒	A description of any assumptions or corrections, such as tests of normality and adjustment for multiple comparisons
☐	☒	A full description of the statistical parameters including central tendency (e.g. means) or other basic estimates (e.g. regression coefficient) AND variation (e.g. standard deviation) or associated estimates of uncertainty (e.g. confidence intervals)
☐	☒	For null hypothesis testing, the test statistic (e.g. *F*, *t*, *r*) with confidence intervals, effect sizes, degrees of freedom and *P* value noted<br>*Give P values as exact values whenever suitable.*
☒	☐	For Bayesian analysis, information on the choice of priors and Markov chain Monte Carlo settings
☒	☐	For hierarchical and complex designs, identification of the appropriate level for tests and full reporting of outcomes
☒	☐	Estimates of effect sizes (e.g. Cohen's *d*, Pearson's *r*), indicating how they were calculated

Our web collection on statistics for biologists contains articles on many of the points above.

Software and code

Policy information about availability of computer code

Data collection	Data were collected using the commercial software, Control FOUR of the WITec Suite (WITec GmbH, Germany).
Data analysis	Data was analyzed in Igor Pro (version 8.0.4.2), using custom code that has been made available on the University of Cambridge's institutional repository Apollo. DOI provided in manuscript.

For manuscripts utilizing custom algorithms or software that are central to the research but not yet described in published literature, software must be made available to editors and reviewers. We strongly encourage code deposition in a community repository (e.g. GitHub). See the Nature Portfolio guidelines for submitting code & software for further information.

Data

Policy information about availability of data

All manuscripts must include a data availability statement. This statement should provide the following information, where applicable:

- Accession codes, unique identifiers, or web links for publicly available datasets
- A description of any restrictions on data availability
- For clinical datasets or third party data, please ensure that the statement adheres to our policy

Raw data and processed data used in the figures has been made available on the University of Cambridge's institutional repository Apollo and DOI provided in the manuscript.

Research involving human participants, their data, or biological material

Policy information about studies with human participants or human data. See also policy information about sex, gender (identity/presentation), and sexual orientation and race, ethnicity and racism.

Reporting on sex and gender	N/A (no human data).
Reporting on race, ethnicity, or other socially relevant groupings	N/A (no human data).
Population characteristics	N/A (no human data).
Recruitment	N/A (no human data).
Ethics oversight	N/A (no human data).

Note that full information on the approval of the study protocol must also be provided in the manuscript.

Field-specific reporting

Please select the one below that is the best fit for your research. If you are not sure, read the appropriate sections before making your selection.

- [x] Life sciences
- [ ] Behavioural & social sciences
- [ ] Ecological, evolutionary & environmental sciences

For a reference copy of the document with all sections, see nature.com/documents/nr-reporting-summary-flat.pdf

Life sciences study design

All studies must disclose on these points even when the disclosure is negative.

Sample size	Sample size was chosen based on the number of cells that could be imaged within 2-3 hours with our set-up, which was regarded as a time span within which cell division and metabolic changes were limited. No sample size was predetermined.
Data exclusions	No data was excluded.
Replication	Repeat measurements on successively cultivated Fh1-KO cl.19 cells were successful. To verify replication, we used two clones of Fh1-KO; cl.1 displayed lower fumarate levels than cl.9 but higher levels than Fh1-WT. Repeat measurements on different sections of mouse tissue were unsuccessful.
Randomization	Randomization was not relevant as no interventions were performed; these studies were intended to measure existing differences in fumarate concentrations.
Blinding	In the mouse tissue studies, the investigator was blinded to group allocation during data collection and analysis.

Reporting for specific materials, systems and methods

We require information from authors about some types of materials, experimental systems and methods used in many studies. Here, indicate whether each material, system or method listed is relevant to your study. If you are not sure if a list item applies to your research, read the appropriate section before selecting a response.

Materials & experimental systems

n/a	Involved in the study	
x		Antibodies
	x	Eukaryotic cell lines
x		Palaeontology and archaeology
	x	Animals and other organisms
x		Clinical data
x		Dual use research of concern
x		Plants

Methods

n/a	Involved in the study	
x		ChIP-seq
x		Flow cytometry
x		MRI-based neuroimaging

Eukaryotic cell lines

Policy information about cell lines and Sex and Gender in Research

Cell line source(s)	We used Fh1-proficient (Fh1-WT) and Fh1-deficient (Fh1-KO) cell lines, both of which are adherent epithelial murine kidney cells originally derived as described in doi.org/10.1038/nature10363. Briefly, in this method, immortalized Fh1-proficient epithelial kidney cells from Fh1 conditional knock out mice are infected with recombinant adenovirus expressing Cre recombinase to target the Fh1 alleles. Two clones of the Fh1-KO cell line were used, termed clone 1 and clone 19. Cells were provided by the laboratory of Christian Frezza, co-corresponding author.
Authentication	Cell lines were not authenticated.
Mycoplasma contamination	All cell lines tested negative for mycoplasma contamination.
Commonly misidentified lines (See ICLAC register)	None.

Animals and other research organisms

Policy information about studies involving animals; ARRIVE guidelines recommended for reporting animal research, and Sex and Gender in Research

Laboratory animals	Mice were of mixed genetic background C57BL/6 and 129/SvJ. Mice were between 10 and 12 weeks old.
Wild animals	No wild animals were used in this study.
Reporting on sex	Sex of the animals was not considered, as this strudy is fundamentally an imaging study (aiming to determine whether fumarate levels can be established in live tissues) rather than an intervention study that investigates the effect on the animals.
Field-collected samples	No field-collected samples were used in the study.
Ethics oversight	All animal experiments were conducted in accordance with the United Kingdom Animals (Scientific Procedures) Act, 1986 (project licence P8A516814).

Note that full information on the approval of the study protocol must also be provided in the manuscript.
