# Supplementary material for: Raman micro-spectroscopy reveals the spatial distribution of fumarate in cells and tissues
Source: Nat Commun. 2024 Jun 25;15:5386. doi: 10.1038/s41467-024-49403-w (PMC11199670; doi:10.1038/s41467-024-49403-w)
Supplement: Supplementary file 3 — Description of Additional Supplementary Files [file 41467_2024_49403_MOESM3_ESM.pdf]

Supplementary Movie 1:
Animation of the C-H deformation (*δ*) mode of fully dissociated fumarate in vacuum, generated by DFT calculation (1261 cm^{-1} band; 1277 cm^{-1} observed experimentally).

Supplementary Movie 2:
Animation of the symmetric CO_2^- stretch (*v*) mode of fully dissociated fumarate in vacuum, generated by DFT calculation (1410 cm^{-1} band; 1401 cm^{-1} observed experimentally).

Supplementary Movie 3:
Animation of the C=C stretch/CO_2^- symmetric bending mode of fully dissociated fumarate in vacuum, generated by DFT calculation (1723 cm^{-1} band; 1652 cm^{-1} observed experimentally).

Supplementary Movie 4:
Animation of the C=C stretch/CO_2^- asymmetric bending mode of fully dissociated fumarate in vacuum, generated by DFT calculation (1670 cm^{-1} band).
