# Supplementary figures and images for: Raman micro-spectroscopy reveals the spatial distribution of fumarate in cells and tissues
Source: Nat Commun. 2024 Jun 25;15:5386. doi: 10.1038/s41467-024-49403-w (PMC11199670; doi:10.1038/s41467-024-49403-w)

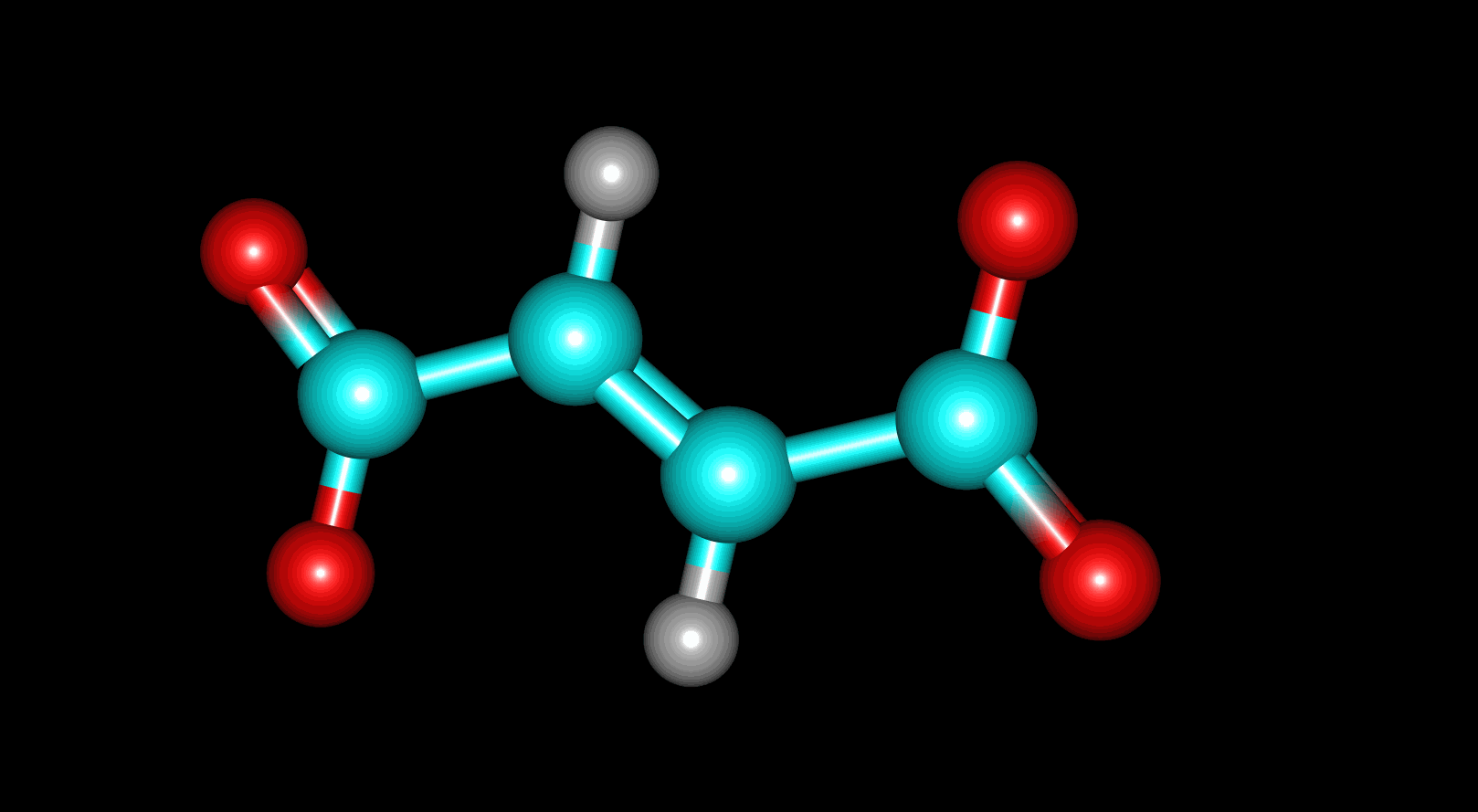

Supplement: Supplementary file 5 — Supplementary Movie 1 [file 41467_2024_49403_MOESM5_ESM.gif]

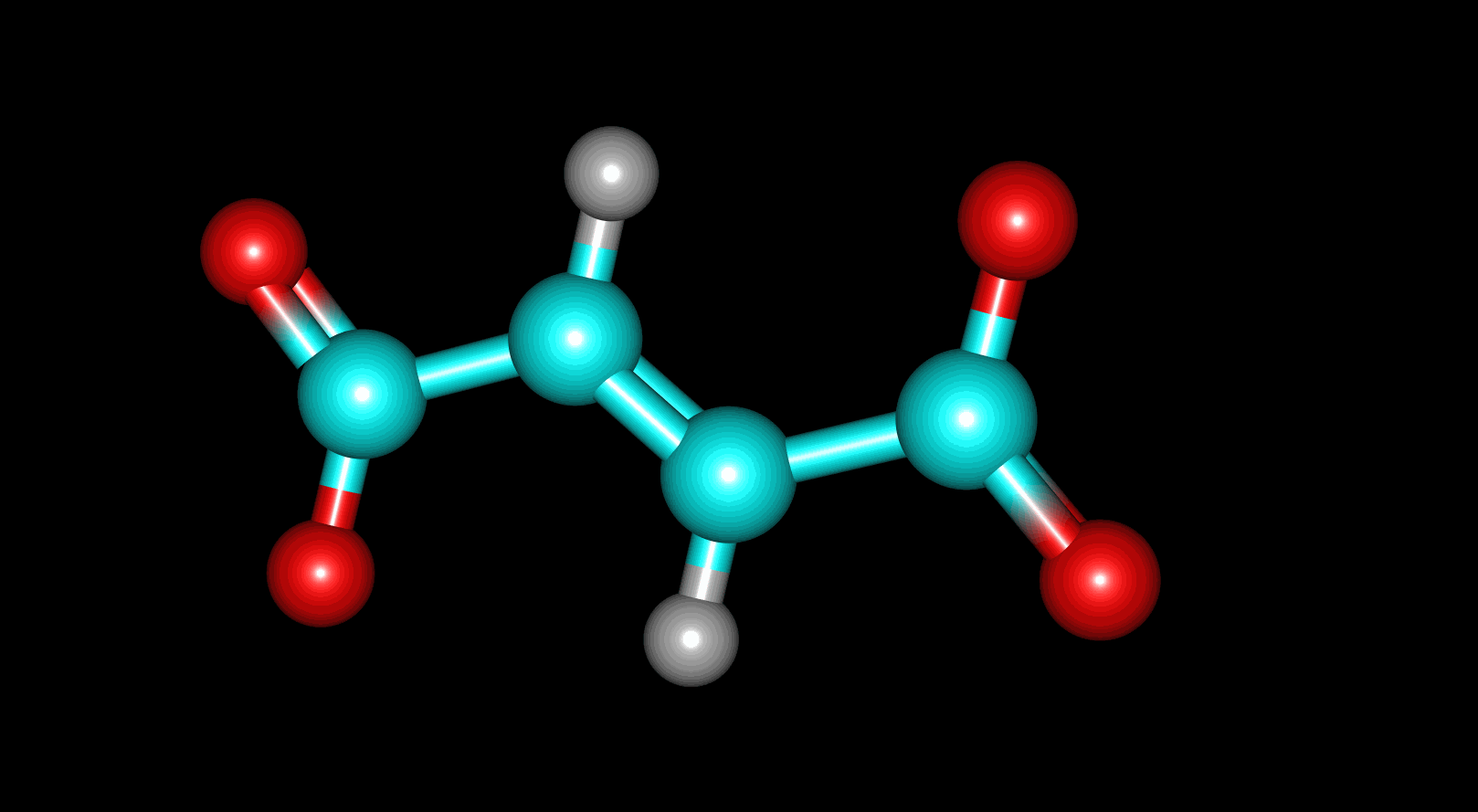

Supplement: Supplementary file 6 — Supplementary Movie 2 [file 41467_2024_49403_MOESM6_ESM.gif]

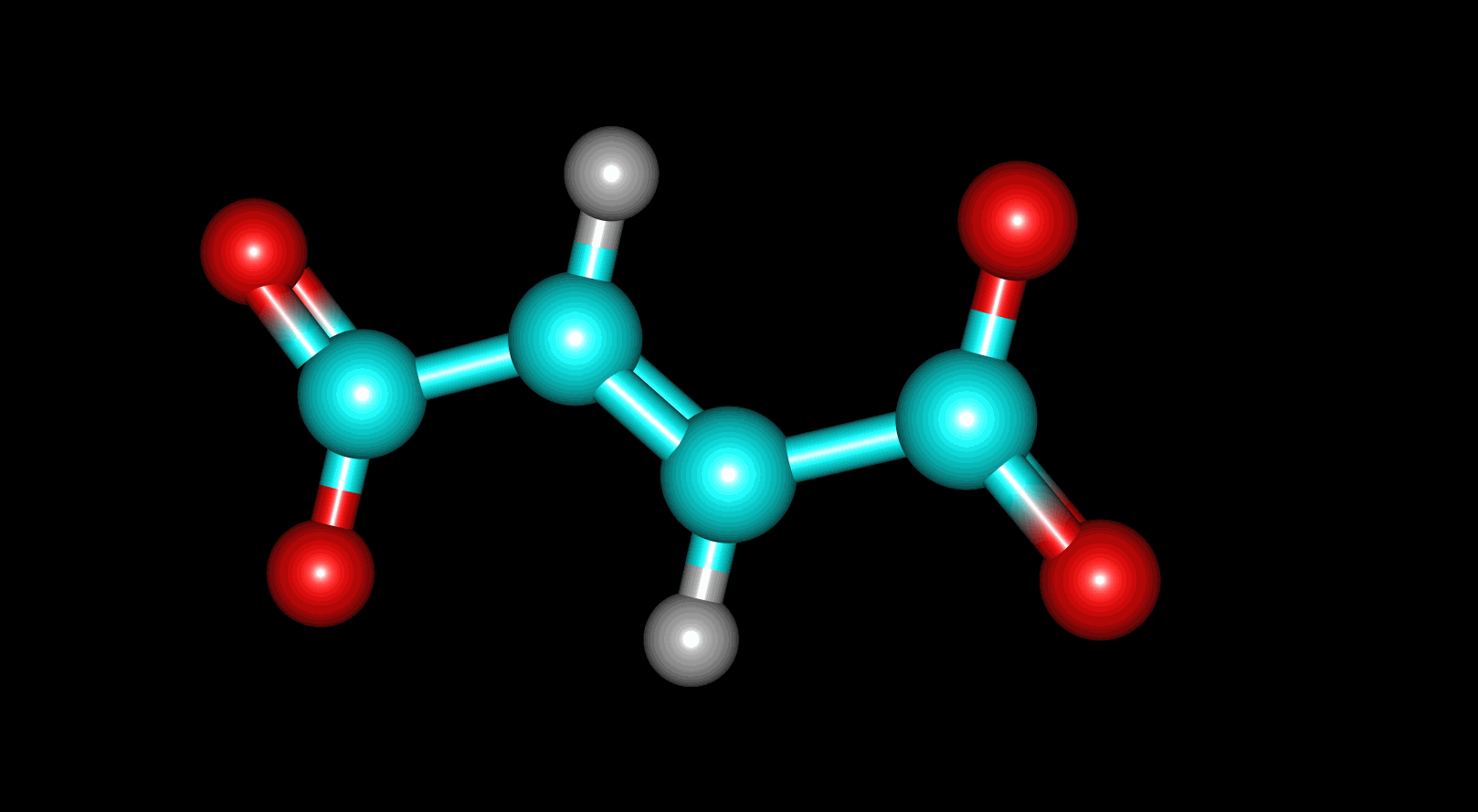

Supplement: Supplementary file 7 — Supplementary Movie 3 [file 41467_2024_49403_MOESM7_ESM.gif]

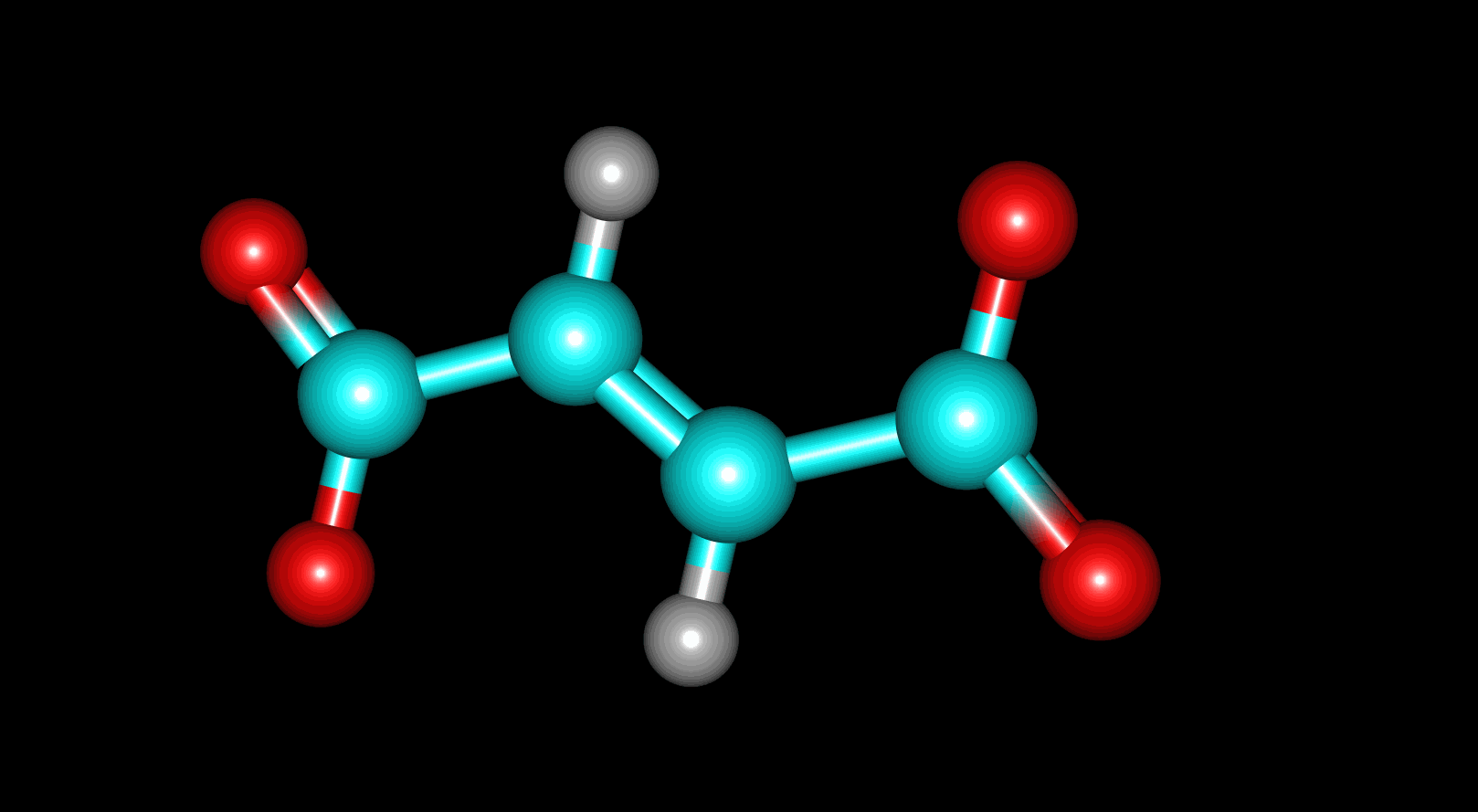

Supplement: Supplementary file 8 — Supplementary Movie 4 [file 41467_2024_49403_MOESM8_ESM.gif]
